# Sensitivity of fluvial sediment source apportionment to mixing model assumptions: A Bayesian model comparison

**DOI:** 10.1002/2014WR016194

**Published:** 2014-11-21

**Authors:** Richard J Cooper, Tobias Krueger, Kevin M Hiscock, Barry G Rawlins

**Affiliations:** 1 School of Environmental Sciences, University of East Anglia, Norwich Research ParkNorwich, Norfolk, UK; 2 IRI THESys, Humboldt UniversityBerlin, Germany; 3 British Geological SurveyKeyworth, Nottingham, UK

**Keywords:** Bayesian, mixing model, fingerprinting, optimization, sensitivity analysis, sediment

## Abstract

Mixing models have become increasingly common tools for apportioning fluvial sediment load to various sediment sources across catchments using a wide variety of Bayesian and frequentist modeling approaches. In this study, we demonstrate how different model setups can impact upon resulting source apportionment estimates in a Bayesian framework via a one-factor-at-a-time (OFAT) sensitivity analysis. We formulate 13 versions of a mixing model, each with different error assumptions and model structural choices, and apply them to sediment geochemistry data from the River Blackwater, Norfolk, UK, to apportion suspended particulate matter (SPM) contributions from three sources (arable topsoils, road verges, and subsurface material) under base flow conditions between August 2012 and August 2013. Whilst all 13 models estimate subsurface sources to be the largest contributor of SPM (median ∼76%), comparison of apportionment estimates reveal varying degrees of sensitivity to changing priors, inclusion of covariance terms, incorporation of time-variant distributions, and methods of proportion characterization. We also demonstrate differences in apportionment results between a full and an empirical Bayesian setup, and between a Bayesian and a frequentist optimization approach. This OFAT sensitivity analysis reveals that mixing model structural choices and error assumptions can significantly impact upon sediment source apportionment results, with estimated median contributions in this study varying by up to 21% between model versions. Users of mixing models are therefore strongly advised to carefully consider and justify their choice of model structure prior to conducting sediment source apportionment investigations.

**Key Points:**

- An OFAT sensitivity analysis of sediment fingerprinting mixing models is conducted
- Bayesian models display high sensitivity to error assumptions and structural choices
- Source apportionment results differ between Bayesian and frequentist approaches

## Introduction

Source apportionment mixing models have been employed across a range of scientific disciplines to estimate the proportions of various sources that feed into a particular mixture or “target” of interest. They are all based on the fundamental assumption that the composition of the target being studied, whether that be hair samples from mammals [*Darimont et al*., 2009] or sediment from rivers [*Thompson et al*., 2013], is a function of the composition of potential sources multiplied by their proportional contribution to the target. This approach relies on selecting appropriate markers or “fingerprints” that can be traced from the source to the target in a reliable manner through well-understood biotic or abiotic pathways. In ecology, stable isotope mixing models (SIMMs) have been used extensively to investigate the dietary intake of organisms by comparing the stable isotopic composition (typically δ^13^C and δ ^15^N ratios) of some part of an organism's body against the isotopically distinct food sources it is believed to consume [*Tarroux et al*., 2012; *Hindell et al*., 2013]. Similarly, within the geosciences, a wide variety of fingerprints, ranging from compound specific stable isotopes [*Fox et al*., 2010; *Puttock et al*., 2012], to fallout radionuclides [*Schuller et al*., 2013; *Slimane et al*., 2013], and major and trace elements [*Evrard et al*., 2013; *Yao et al*., 2013], have all been used to estimate the contribution of various terrestrial sediment sources to fluvial sediment load.

The ability of any mixing model to accurately represent source contributions to a mixture will ultimately be determined by the error assumptions and model structural choices made by the modeler. Two overarching statistical approaches are commonly employed in model formulation. The first is traditional Maximum Likelihood optimization which has been widely used in sediment fingerprinting studies for the past 15–20 years [*Gruszowski et al*., 2003; *Walling et al*., 2003; *Martínez-Carreras et al*., 2010; *Walling*, 2013]. These frequentist models commonly minimize the sum of squared residuals as outlined by *Collins et al*. [1997], with more recent approaches typically coupling parameter optimization with Monte Carlo based stochastic sampling to represent uncertainties associated with source area and target sediment variability [*Collins et al*., 2013; *Wilkinson et al*., 2013]. However, these models are often inconsistent in their uncertainty representation and they lack the structural flexibility to coherently translate all sources of error into model results. Consequently, Bayesian mixing models have come to increasing prominence over the last 5–10 years as a more robust alternative for comprehensively incorporating uncertainty into models [*Fox and Papanicolaou*, 2008; *Rowan et al*., 2011; *Massoudieh et al*., 2012; *D'Haen et al*., 2013; *Dutton et al*., 2013; *Nosrati et al*., 2014]. Fundamentally, the Bayesian approach is advantageous over frequentist methods as it enables all known and residual uncertainties associated with the mixing model and the data set to be coherently translated into parameter probability distributions in a hierarchical framework. A wide variety of Bayesian model setups have been employed, with previous studies differing in the choice of prior parameter distributions, the inclusion of covariance terms, the incorporation of time-variant distributions, the methods of proportion characterization, and whether full or empirical Bayesian formulations are used. The purpose of this study was to ascertain the sensitivity of source apportionment estimates to these variations in mixing model structure. We conducted a one-factor-at-a-time (OFAT) sensitivity analysis of 13 model versions, each with at least one differing structural element. All 13 versions were applied to suspended particulate matter (SPM) geochemistry data from the River Blackwater, Norfolk, UK, to apportion sediment contributions from three different source areas under base flow conditions.

## Methods

### M1: The Benchmark Model

**Figure 1:**
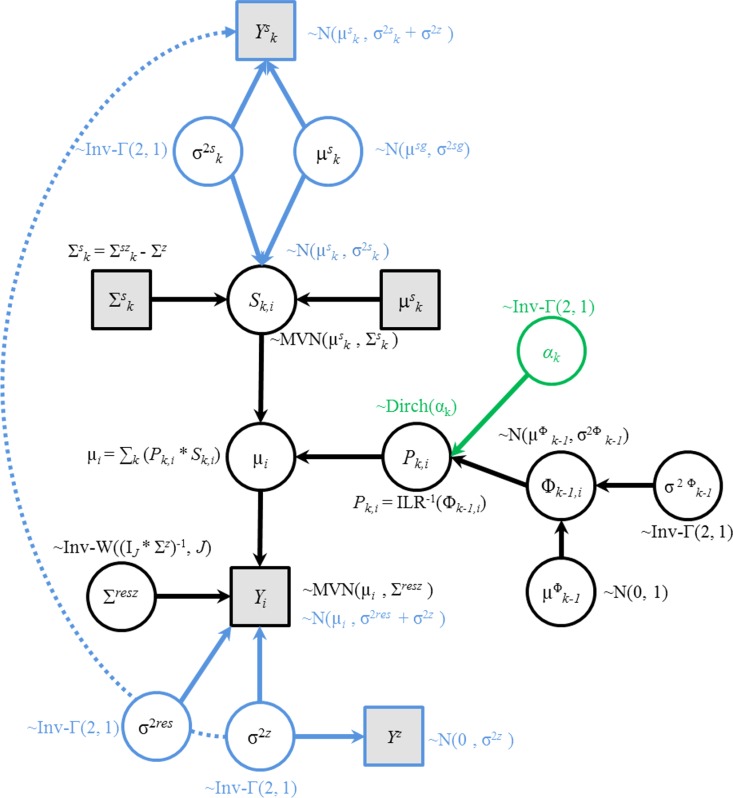
A Directed Acyclic Graph (DAG) of the benchmark Bayesian mixing model (M1) in black, with extension to the Dirichlet distribution parameterization in green (M11) and the full Bayesian model (M12) in blue. Gray squares indicate nodes with observed data, whilst white circles indicate random variables estimated by the Markov Chain Monte Carlo (MCMC) procedure. Prior distributions and deterministic link equations are noted alongside. See text for symbol meanings.

Mixing model 1 (M1) represents our so called “benchmark” Bayesian model against which the other 12 versions were compared. This empirical Bayesian mixing model represents a modified version of that developed by *Parnell et al*. [2013] for quantifying the dietary intake of geese. The model follows Bayes' theorem:


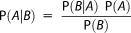
(1)

which states that the probability (P) of *A* given *B* (P(*A*|*B*) – the posterior) is a function of prior belief in *A* (P*(A)*) and a quotient that represents the support knowledge of *B* provides to *A* (P*(B|A)/*P*(B)*). This model is succinctly summarized by the Directed Acyclic Graph [DAG; *Lunn et al*., 2000] in Figure 1, which links together sets of random variable parent nodes with their conditional child node dependencies. Symbol meanings are as follows: *Y* is the measured concentration of fingerprints in SPM; *Y^s^* and *S* are the measured and modeled concentrations of fingerprints in source area sediments, respectively; *P* and Φ are the sediment contributions of each source area in original and ILR-transformed space (see below); µ*^sg^* and σ^2^*^sg^* are a priori guesses at the hyperparameters of the source means; *Y^z^* is the measured instrument error; *j* and *k* are the fingerprint and source indices, respectively; *J* is the number of fingerprints; ∑ are covariance matrices; σ^2^ are variances; µ are means; *i* is the model time step index; and MVN, N, Dirch, Inv-W, and Inv-Γ represent multivariate normal, normal, Dirichlet, inverse multivariate Wishart, and inverse gamma distributions, respectively.

The core model formula is a mass balance whereby the concentration of each fingerprint in SPM (*Y*) is derived from the concentration of that fingerprint in each source area (*S*) multiplied by the proportional sediment contribution (*P*) from that source. The likelihood function is, accordingly:


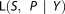
(2)

which essentially asks “what is the likelihood of *S* and *P* given our knowledge of *Y*?” The solution is solved via a Markov Chain Monte Carlo (MCMC) sampling procedure of the full parameter distributions.

Prior distributions for the sources (*S*) are estimated via an empirical Bayesian approach, whereby MVN distributions are parameterized using the actual measured means (µ^s^) and covariance matrices (Σ^s^) of fingerprints in all source area samples. For the prior probability on the proportions (*P*), the procedure of *Parnell et al*. [2013] was adopted by applying a geometric transformation to the data—in this instance the isometric log-ratio (ILR) transform [*Egozcue et al*., 2003]. Transforming the compositional data in this way ensures that all proportions are independent (orthogonal) in transformed space on the complete real scale, thus allowing univariate normal priors, while all proportions are positive and sum to unity in the original space. The ILR transformation is specified as follows:


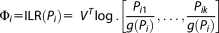
(3)

where *V* is a *k-1 x k* triangular Helmert matrix and *g*(*P*_i_) is the geometric mean. The reverse transformation of Φ to return real *P* values occurs by exponentiation and re-normalization [*Egozcue et al*., 2003]:


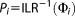
(4)

The Φ values are estimated by prior hyperparameter distributions of µ^Φ^ and σ ^2Φ^ that are assigned weakly informative normal and inverse gamma distributions, respectively. Combined instrument precision and residual error (Σ*^resz^*) was incorporated into the model via a semi-informative, inverse Wishart distribution—the conjugate prior of the MVN [*Sun and Berger*, 2006]. Here, the Wishart scale matrix (Ω) is represented by the product of an uninformative *JxJ* identity matrix (*I_J_*) for residual error, and an informative covariance matrix (Σ*^z^*) for instrument error. Σ*^z^* was derived empirically from 42 repeat analyses of a sediment standard. Inclusion of the residual error term accounts for uncertainties not explicitly incorporated into the model.

The complete Bayesian posterior distribution can be written in condensed form as:


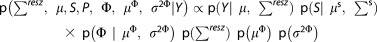
(5)

### OFAT Sensitivity Analysis

Twelve variants to the benchmark model were formulated (Table 1). Models 2–5 (M2–M5) assess the impact of altering the mean and variance hyperparameter terms for the prior proportion distributions. Models 6–8 (M6–M8) evaluate modifications in covariance structure. Model 9 (M9) considers changes in the temporal variability of source distributions. Models 10 (M10) and 11 (M11) assess the impact of proportion characterization. Model 12 (M12) contrasts the empirical with the full Bayesian approach. And finally, model 13 (M13) assesses the frequentist optimization technique.

**Table 1:** Comparison of the Structure and Parameters of the 13 Different Mixing Model Formulations^a^

Model Version	Inference	Full or Empirical Bayes	Source Distribution (*S*)	Source Covariance (Σ*^s^*)	Residual/Instrument Covariance (Σ^resz^)	Proportion Method (*P*)	Proportion Mean (µ^Φ^)	Proportion Variance (σ^2Φ^)
M1	Bayesian	Empirical	[*i*,*k*]	Yes	Yes	ILR	∼N(0,1)	[k] ∼Inv-Γ(2,1)
M2	Bayesian	Empirical	[*i*,*k*]	Yes	Yes	ILR	∼N(0,1)	**[k] ∼Inv-Γ(0.001,0.001)**
M3	Bayesian	Empirical	[*i*,*k*]	Yes	Yes	ILR	**∼N(0,1000)**	[k] ∼Inv-Γ(2,1)
M4	Bayesian	Empirical	[*i*,*k*]	Yes	Yes	ILR	**0**	[k] ∼Inv-Γ(2,1)
M5	Bayesian	Empirical	[*i*,*k*]	Yes	Yes	ILR	∼N(0,1)	**∼Inv-Γ(2,1)**
M6	Bayesian	Empirical	[*i*,*k*]	**No**	Yes	ILR	∼N(0,1)	[k] ∼Inv-Γ(2,1)
M7	Bayesian	Empirical	[*i*,*k*]	Yes	**No**	ILR	∼N(0,1)	[k] ∼Inv-Γ(2,1)
M8	Bayesian	Empirical	[*i*,*k*]	**No**	**No**	ILR	∼N(0,1)	[k] ∼Inv-Γ(2,1)
M9	Bayesian	Empirical	**[*k*]**	Yes	Yes	ILR	∼N(0,1)	[k] ∼Inv-Γ(2,1)
M10	Bayesian	Empirical	[*i*,*k*]	Yes	Yes	**CLR**	∼N(0,1)	[k] ∼Inv-Γ(2,1)
M11	Bayesian	Empirical	[*i*,*k*]	Yes	Yes	**Dirichlet**	N/A	N/A
M12	Bayesian	**Full**	[*i*,*k*]	**No**	**No**	ILR	∼N(0,1)	[k] ∼Inv-Γ(2,1)
M13	**Frequentist**	N/A	**[*k*]**	Yes	Yes	N/A	N/A	N/A

a Differences from the benchmark model (M1) are emphasized in bold. ∼N and ∼Inv-Γ refer to normal and inverse gamma distributions, respectively. Square brackets denote distributions or parameters that vary with each source [*k*] or time step [*i*].

#### Hyperparameters

Hyperparameters are an important component of Bayesian inference and are one of the main differences to the frequentist approach. By setting informative hyperparameters on model priors one is able to incorporate prior knowledge of the system into the model, whilst setting uninformative hyperparameters allows the modeler to relax assumptions that the system being modeled has been fully understood [*Fox and Papanicolaou et al*., 2008]. Here, we tested mixing model sensitivity to changing hyperparameter distributions on the transformed proportions (Φ). In M1, the mean (µ^Φ^) and variance (σ^2Φ^) parameters of Φ were assigned normal and inverse gamma distributions of N(0, 1) and Inv-Γ(2,1), respectively, where the Inv-Γ employs a shape-rate parameterization and ensures positivity [*Plummer*, 2003]. In M2, Φ was assigned a more informative distribution through the selection of a narrower inverse-gamma distribution on the variance (σ^2Φ^∼Inv-Γ(0.001,0.001)). In M3, Φ was assigned a less informative distribution through a wider normal distribution on the mean (µ^Φ^ ∼N(0,1000)). In M4, µ^Φ^ was fixed to zero which equates to a more rigid prior assumption of 33.33% mean contribution from each source [e.g., *Parnell et al*., 2013]. And finally, a common σ^2Φ^ for all sources (*k*) was tested in M5 [e.g., *Hopkins and Ferguson*, 2012].

#### Covariance Terms

Covariation between the geochemical properties of soils and sediments is well known [e.g., *Rawlins*, 2011]. Incorporating correlation between input parameters into models is also known to have considerable implications for estimated uncertainties, thus making it an important part of both frequentist and Bayesian inference [*Dilks et al*., 1992; *Smith et al*., 1992]. This was achieved in M1 through the use of MVN distributions to parameterize source and target variability (Σ*^s^* and Σ*^resz^*), with an inverse-Wishart prior on the combined residual and instrument error term (Σ*^resz^*). Model sensitivity to the inclusion of covariance terms was then assessed by first removing covariation between source area fingerprints (M6 and M8) and then removing it from the combined residual and instrument error term (M7 and M8) by setting the off-diagonal elements of these covariance matrices to zero. For M7 and M8, this involved replacing the inverse-Wishart prior on Σ*^resz^* with an inverse-gamma prior Inv-Γ(2,1) on the variance terms (the diagonal elements of Σ*^resz^*).

#### Time-Variable Sources

In M1, data are drawn from new source distributions (*S*) at each time step, thereby enabling temporal variability in sediment source geochemistry to be incorporated into the model. This is important because it allows the model to implicitly account for the erodability and connectivity of different locations within any given source classification [*Fox and Papanicolaou et al*., 2008], whilst also enabling the model to account for transient sediment storage within the fluvial system. We assessed model sensitivity to the inclusion of temporal source variability by removing the temporal component in M9, thus ensuring source area distributions were kept constant for each SPM sample. Other researchers [e.g., *Brewer et al*., 2011; *Parnell et al*., 2013] have included more explicit temporal source components in their mixing models, such as using splines to model autocorrelation within the data set. These features can allow specific knowledge of temporal relationships to be incorporated into the model (e.g., specific bed sediment storage parameters). This aspect represents a promising area of mixing model development which warrants separate investigation and, as such, was not explored further here.

#### Characterizing Proportions

Logically, the posterior distribution of the proportions should conform to positivity and unity requirements, such that contributions from any one source must be between zero and one, and the contribution from all sources must sum to one. Such assumptions can be met through the selection of appropriate parameterizations of *P*. As discussed above, this is achieved in M1 through the ILR transformation. Another transformation for compositional data is the centered log-ratio transformation (CLR) [*Aitchison*, 1986], tested in M10, which has been applied in previous mixing model studies [*Semmens et al*., 2009; *Hopkins and Ferguson*, 2012]. The CLR is defined as:


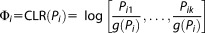
(6)

where *g*(*P_i_*) is the geometric mean of the proportions. An alternative to transformation is to use a Dirichlet prior on the untransformed proportions (M11). Being a multivariate generalization of the Beta distribution, the Dirichlet is defined on the interval [0, 1] in the simplex and therefore conforms to the positivity and unity requirements [*Lingwall et al*., 2008]. The Dirichlet shape parameters (α) were assigned weakly informative hyperpriors (Inv-Γ(2,1)).

#### Full Versus Empirical Bayes

The distinguishing feature of empirical Bayesian approaches (M1) is that some prior distributions are estimated offline using deterministic data, meaning that the parameters of the prior distributions are essentially fixed at the Maximum Likelihood estimate [*Carlin and Louis*, 1996]. This has the advantage of reducing model complexity and correlation between parameters; however, it also reduces model flexibility and can lead to biased estimates where the data are unrepresentative—a particular problem with small sample sizes (e.g., <20) [*Ward et al*., 2010]. The alternative is a full Bayesian approach [*Palmer and Douglas*, 2008; *Hopkins and Ferguson*, 2012] where hyperparameters are themselves treated as random variables with prior distributions and all priors are integrated out during the numerical solution [*Fox and Papanicolaou et al*., 2007, 2008]. This is true to the Bayesian paradigm, whereas empirical Bayes is an approximation for numerical tractability. We tested the sensitivity to a full Bayesian approach in M12, where the prior means of the sources (*S*) were assigned informative N(µ^sg^,σ^2sg^) distributions to aid convergence, while weakly informative Inv-Γ(2,1) distributions were assigned to the source variances (Figure 1). Covariation was omitted from the full Bayesian formulation due to numerical difficulties in ensuring all covariance matrices met the required positive-definiteness criteria. The empirical Bayes equivalent of M12 is thus M8.

#### Frequentist Models

Bayesian mixing models and frequentist optimization differ fundamentally in the way they use probability. In Bayesian models, the parameters are treated as unknown random variables and are determined probabilistically [*Lunn et al*., 2000]. In frequentist approaches, the model parameters are deterministic and only their estimates are random [*Carlin and Louis*, 1996]. To understand the impact on source apportionment of selecting Bayesian or frequentist approaches, we compared M1 against M13—a modified version of the least squares regression sediment fingerprinting model presented by *Collins et al*. [[Bibr b6],[Bibr b8]]. The model solution was determined by optimization through minimizing the sum of squared residuals (SSR):


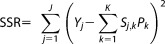
(7)

whilst satisfying the following constraints:


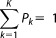


and


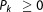


Similar to *Collins et al*. [2013], source and target variability were incorporated by nesting the optimization step within an ordinary Monte Carlo iteration that sampled from the source and target distributions. As with the Bayesian approach, but in contrast to *Collins et al*. [2013], source area fingerprints (*S*) were parameterized via MVN distributions to account for covariance. Instrument precision, as determined via repeat analysis of sediment standards, was incorporated via the covariance parameter of the MVN distribution used to parameterize the target (*Y*) values. Following standard practice, the robustness of the source apportionment estimates was evaluated via the goodness-of-fit (GOF) criterion presented by *Martínez-Carreras et al*. [2010] (equation 8), with a tolerance criterion for acceptance set to >0.95, as recommended by *Motha et al*. [2003]:


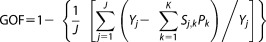
(10)

This approach also differs from that presented by *Collins et al*. [2013] in that no particle size or organic matter correction factors were incorporated within the model. Such corrections were omitted from both the frequentist and Bayesian models because recent research [*Smith and Blake*, 2014] has found that generalizing the relationships between organic matter, particle size, and sediment geochemistry is complex and carries the inherent risk of overcorrecting the data and thereby generating additional unknown levels of uncertainty which may bias the results. Furthermore, as *Smith and Blake* [2014] highlight, differences in particle size and organic matter content between source area sediments and target SPM are unlikely to reflect solely downstream selective transport. Some of this variation likely reflects genuine differences between the source groups, and thus applying corrections would remove a potentially helpful discriminatory characteristic.

### Data Collection

**Figure 2:**
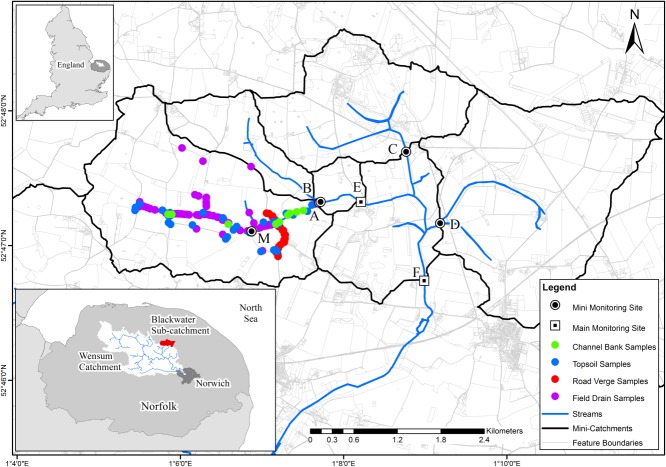
The Blackwater subcatchment of the River Wensum, UK, showing the locations of the source area sampling, the six minicatchments (A-F) and the seven bankside monitoring stations that form the field installations of the River Wensum DTC project. This research was focused within minicatchment A.

Sediment geochemistry data were collected as part of the UK River Wensum Demonstration Test Catchment (DTC) project, for which comprehensive details of the study site, fieldwork, and laboratory procedures can be found in *Cooper et al*. [2014b]. To summarize, this study focused on a 5.4 km^2^ portion (minicatchment A) of the lowland Blackwater subcatchment of the River Wensum, Norfolk, UK (Figure 2). The catchment bedrock is Cretaceous White Chalk at a depth of ∼20 m, overlain by superficial deposits of Mid-Pleistocene diamicton glacial tills (0.2–20 m depth) and interspersed with layers of glaciofluvial and glaciolacustrine sands and gravels. The principal soil types are clay loam to sandy clay loam to a depth of at least 0.2 m [*Hiscock*, 1993; *Lewis*, 2011]. Ninety-two percent of the catchment is under intensive arable cultivation, with 5% under grassland, 2% woodland, and 1% rural settlements. Three potential sediment contributing source areas where identified across the subcatchment, namely, arable topsoils, damaged road verges, and a combined stream channel bank and agricultural field drain “subsurface” source. Previous studies have demonstrated that merging sources with similar properties, as was true here for channel banks and field drains, can significantly improve source apportionment performance [*Parnell et al*., 2010; *Ward et al*., 2011]. Thirty samples of both topsoil and road verge material were collected as <50 mm surface scrapes from areas susceptible to erosion that had potentially high connectivity to the stream channel. Channel bank sediments were sampled as surface scrapes at depths of 10, 30, and 50 cm above the streambed at 10 locations along a 2.9 km stretch of the river to yield 30 samples. Sediments discharging from field drains were collected by bulking together grab samples taken from 120 drains identified across the catchment to yield 30 samples for analysis. For the target mixture data, instream SPM was collected at monitoring kiosk A as 6 L grab samples under base flow conditions at 1–2 week intervals between 7th August 2012 and 6th August 2013, yielding a total of 40 samples.

**Figure 3:**
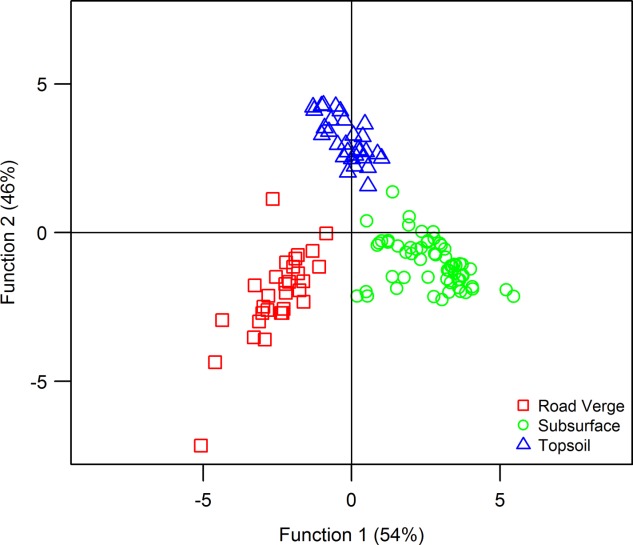
Discriminant function analysis plot of source area sediments.

All SPM samples were vacuum filtered through 0.45 µm Millipore quartz fiber filter (QFF) papers to extract particulate matter. Consolidated source area material was first sonicated for 7 min in a water bath and wet sieved to sub-63 µm before being vacuum filtered onto QFF papers to ensure that particle size distributions, and thus geochemistry, of sources and SPM were comparable [*Horowitz and Elrick*, 1987]. The geochemistry of all the sediment covered QFF papers was then assessed directly by X-ray fluorescence spectroscopy (XRFS) following the methods of *Cooper et al*. [2014a], yielding concentrations for 11 major elemental fingerprints. The Kruskal-Wallis H-test and stepwise discriminant function analysis (DFA) where used to determine which fingerprints could successfully differentiate between source areas (Figure 3). The resultant suite of eight elements (Al, Ca, Ce, Fe, K, Mg, Na, and Ti) was selected for the mixing models (Table 2). Previous research has demonstrated that, provided fingerprints are legitimate, maximizing the number of tracers can help to significantly improve differentiation and reduce model uncertainties [*Small et al*., 2002; *Parnell et al*., 2010]. In this respect, the DFA tracer reduction step is not strictly necessary. However, we employed it here in order to improve model convergence by reducing the number of variables present, whilst still maintaining sufficient data to facilitate discrimination.

**Table 2:** Summary Geochemistry Data for SPM and Source Area Sediments

Source Areas	Statistic	Concentrations (Weight %)							

		Al	Ca	Ce	Fe	K	Mg	Na	Ti
SPM	Mean	8.17	22.51	0.0051	8.08	1.43	0.70	0.23	0.47
*(n=40)*	SD	0.57	1.76	0.0003	0.68	0.09	0.04	0.02	0.03
Channel banks	Mean	6.97	35.47	0.0036	5.04	1.19	0.61	0.19	0.45
*(n=30)*	SD	2.34	7.65	0.0013	1.65	0.44	0.18	0.06	0.09
Field drains	Mean	6.89	17.50	0.0049	8.21	1.12	0.51	0.26	0.38
*(n=30)*	SD	2.49	8.23	0.0015	5.14	0.39	0.17	0.09	0.11
Road verges	Mean	10.40	6.63	0.0086	6.12	2.08	1.01	0.48	0.61
*(n=30)*	SD	0.99	1.32	0.0007	0.48	0.11	0.09	0.05	0.02
Top soils	Mean	14.07	3.97	0.0091	6.93	2.45	0.88	0.41	0.66
*(n=30)*	SD	1.17	2.00	0.0008	0.62	0.23	0.07	0.04	0.02

### Running the Models

All Bayesian modeling was carried out using the open source software JAGS version 3.3.0 (Just Another Gibbs Sampler) [*Plummer*, 2003] within the R environment [*R Development Core Team*, 2013]. JAGS performs hierarchical Bayesian inference using a Gibbs sampling Markov Chain Monte-Carlo (MCMC) algorithm on the prior probability distributions and the likelihood function to estimate the posterior distribution. All mixing models were run for 750,000 iterations, with a 100,000 sample burn-in and jump length of 225 to minimize autocorrelation between runs. To confirm convergence of the MCMC random walk on the equilibrium distribution, three MCMC chains were run in parallel from different starting conditions and trace plots of the parameter distributions were inspected for mixing. Convergence diagnostics were performed via the “coda” R package [*Plummer et al*., 2006]. The frequentist M13 was run using the “limSolve” R package [*Van den Meersche et al*., 2009] and was afforded 750,000 iterations to converge on the optimum solution.

## Results

### Apportioning Sources of SPM

**Figure 4:**
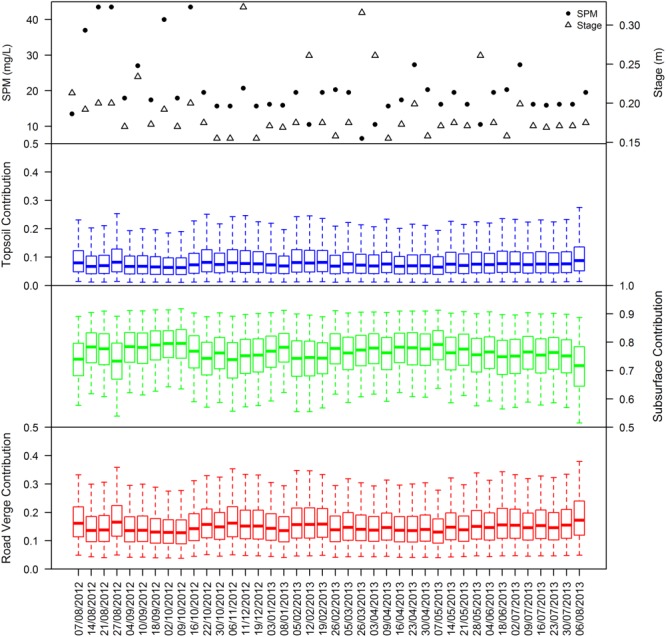
Benchmark model (M1) source apportionment estimates under base flow conditions for the period August 2012 to August 2013. The solid central line, boxes, and whiskers represent the median, 50% and 95% Bayesian credible intervals, respectively.

The source apportionment estimates of M1 reveal subsurface material to be the dominant source of SPM under base flow conditions throughout the period from August 2012 to August 2013 (Figure 4). Estimated median sediment contributions derived from the subsurface source areas vary between 71 and 80% (51–92% at the 95% credible interval), with median contributions of 6–9% (1–27%) for arable topsoils and 12–17% (4–38%) for road verges. In comparison, a median 63% (44–80%) of SPM was estimated to be derived from surface sources during numerous autumn and winter precipitation events at the same location [*Cooper et al*., 2014b]. The dominance of subsurface sediment contribution, particularly during the summer months when field drains cease flowing, indicates that erosion of the lower section of stream channel banks is the primary mechanism of SPM generation under base flow conditions. Relatively low contributions from topsoils and road verges indicate limited surface land-to-river sediment transfer outside of heavy precipitation events, as intuitively would be expected. With negligible surface runoff occurring, the continued contribution of road verge and topsoil material to SPM indicates the resuspension of material from these sources deposited on the streambed during prior precipitation events. Temporal fluctuations in this surface source contribution, which are not correlated to either stage or SPM concentration (Figure 4), likely reflects both the degree of bed disturbance prior to sampling and the antecedent sediment delivery conditions—i.e., whether a rainfall event had delivered topsoil and road verge material to the stream in the days preceding sample collection [*Cooper et al*., 2014b].

### Model Sensitivity

**Figure 5:**
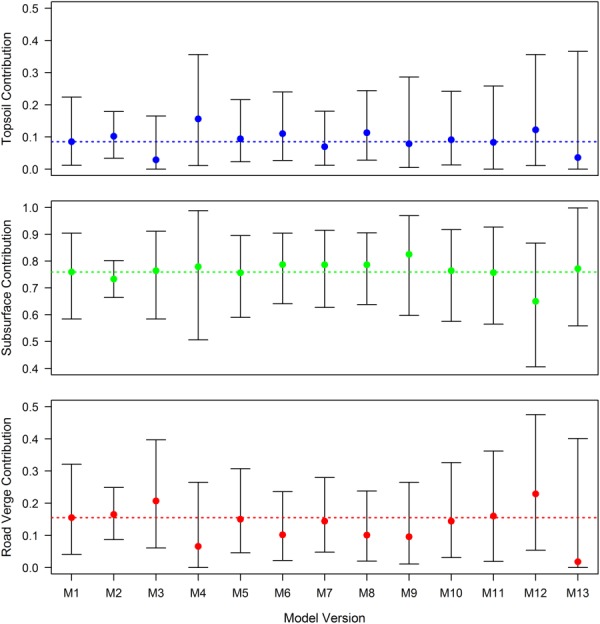
Comparison of SPM source apportionment as estimated by 13 mixing model versions. Results for each version are displayed as the temporal apportionment average across all 40 SPM samples spanning August 2012 to August 2013. Points represent median contributions with associated 95% credible intervals, whilst dashed lines represent the median contribution estimated by M1.

Source apportionment results for the 13 model versions are summarized in Figure 5 as the temporal apportionment average across all 40 SPM samples. Whilst all models estimate subsurface sediments to be the dominant source of SPM in the River Blackwater, significant differences exist in the median contributions and width of credible intervals (CI). These departures from the benchmark model results are explored in turn. Note, depending upon the shape of the posterior distributions, the median contributions across sources do not necessarily sum to unity.

*Models 2–5*: Selection of a more informative hyperparameter distribution for the variance on Φ in M2 had a major impact upon estimated uncertainties, reducing CI widths by 6.7%, 18.2%, and 11.8% for topsoils, subsurface sources, and road verges, respectively, relative to the benchmark model. The impact upon the median source contributions was less pronounced, varying by <2.6% across all sources. The reverse situation arises with M3, where the selection of a vague prior hyperparameter distribution for the mean on Φ resulted in a reduction of estimated median topsoil contribution by 5.6%, while subsurface material and road verge contributions increased by 0.5% and 5.2%, respectively. The range of 95% CIs were also impacted, although not as strongly as for M2. For M4, fixing the prior mean of Φ at zero resulted in some of the largest deviations in median apportionment, with increases of 7.1% and 2.0% estimated for topsoils and subsurface material, respectively, and a decline of 8.9% in estimated road verge contribution. This reversed the order of importance of topsoil and road verge sediment contributions. M4 topsoil and subsurface source CI widths were also ∼15% wider compared with the benchmark model. Last, fixing the variance parameter of M5 across sources had limited impact on model results, with median contributions and CI ranges varying by less than 0.9% and 1.9%, respectively, across all sources.

*Models 6–8*: Omitting source covariance from M6 resulted in a 2.8% and 2.5% increase in estimated median subsurface source and topsoil contributions, respectively, whilst the road verge contribution declined by 5.3%. Additionally, the 95% CI ranges decreased by 5.6% and 6.6% for subsurface and road verges, respectively. Removing covariance from the combined residual and instrument error term (M7) had a less pronounced effect, with median proportions varying by <2.7% across all sources and CI ranges varying by a maximum of 4.8%. Removal of all covariance (M8) impacted most strongly upon median road verge contribution, which declined by 5.4%, and subsurface CI width, which narrowed by 6.2%—overall similar to the behavior of M6, indicating that the mixing model results are more sensitive to the parameterization of source covariance than of instrument and residual covariance.

*Model 9*: Making the source distributions time-invariable significantly increased apportionment uncertainty for both subsurface (5.1%) and topsoil (6.8%) sources. Estimated median proportions were also affected, increasing by 6.6% for the subsurface source and declining by 5.9% and 0.6% for road verges and topsoils, respectively.

*Models 10–11*: Application of the CLR transformation (M10) had only minor influence on the posterior distributions relative to M1. Median proportions varied by less than 1.1% across all sources, whilst the CI ranges increased across all sources by a maximum of 2.1%. Similarly, application of the Dirichlet distribution (M11) also had limited impact on the median proportions which varied by less than 0.5%. CI ranges did, however, increase for all sources by up to 6.2%.

*Model 12*: The full Bayesian model had the greatest impact upon estimated uncertainties relative to M1, increasing CI widths by 13.2%, 14.1%, and 14.2% for topsoil, subsurface sources, and road verges, respectively. Estimated median contributions were also significantly impacted, reducing by 10.9% for subsurface sources and increasing by 3.7% and 7.4% for topsoils and road verges.

*Model 13*: Median subsurface source apportionment of the frequentist model differed by just 1.3% compared to M1, despite the major differences in model structure. However, topsoil and road verge contributions were heavily impacted, declining by 4.9% and 13.7%, respectively, and having strongly positively skewed distributions (Figure 5). Additionally, the 95% CIs were considerably wider, increasing by 15.4%, 12.0%, and 12.1% for topsoil, subsurface, and road verge sources, respectively.

### Model Runtimes

Runtimes for Bayesian models 1–11 were all comparable at between 289 and 402 s to complete 750,000 iterations of each model time step. Relative to the benchmark model (393 s), M9 had the shortest runtime (289 s), reflecting that, with source distributions being time-invariable, fewer nodes had to be modeled. Similarly, the runtime for M11 (345 s) was reduced due to the inclusion of fewer nodes in the parameterization of the proportions. Setting less informative priors on the proportions (M3) slightly increased runtimes (403 s) as the MCMC procedure had to explore a larger parameter space. A significantly longer runtime was recorded for the full Bayesian procedure (M12, 1915 s) due to the greater number of hyperparameters that had to be estimated. The runtime of the frequentist optimization (M13, 429 s) did not differ significantly from that of the benchmark model.

## Discussion

### Hyperparameters

It is generally understood that the more data that are entered into Bayesian models, the less weight the choice of prior hyperparameters will have on the resulting posterior distributions [*Van den Meersche et al*., 2008]. Despite the number of fingerprints (eight) included in our model being greater than that commonly used in source apportionment studies [e.g., *Fox and Papanicolaou et al*., 2008], it was still relatively small compared to the number of parameters that had to be estimated. As a result, the model results demonstrate considerable sensitivity to the choice of hyperparameter values. The narrower hyperparameter distribution used in M2 essentially states that the modeler has greater prior certainty about the shape the posterior distribution should take, hence the reduction in CI width. Similarly, the shifts in posterior median sediment contributions for M3 intuitively make sense, because the selection of a wider prior normal distribution to parameterize the mean on Φ has afforded the posterior proportion distributions greater flexibility to vary over a wider range of possible values. However, not all proportions respond in the same direction due to codependencies and interactions between the parameters. In M4, the reversal in the order of importance for topsoil and road verge contributions highlights that these two sources occupy a similar source geometry in mixing space. This allows for a wider range of possible model solutions, thereby making differentiation difficult and highly dependent upon prior specification. The increased CIs of M4 seem to reflect that as the proportions are pulled toward an unrealistic range around 33.33% a priori, the model is then uncertain where to move next given the limited information content of the data. In fact, all the results appear to demonstrate that sources with high data information content (e.g., subsurface sources clearly distinguished by a strong calcium signature) are less affected by the choice of prior. Overall, these results correspond with the findings of other mixing model studies [e.g., *Moore and Semmens*, 2008; *Franco-Trecu et al*., 2013], and strongly imply that where hyperparameters are to be used, they must be carefully selected to prevent model output being biased by poorly chosen priors. With this in mind, and staying true to the Bayesian paradigm, we recommend using uninformative hyperparameters were possible to reduce biases. However, it is acknowledged that a balance must be struck between setting overly informative priors that may impart bias onto results if the system being modeled is not fully understood, and setting overly vague priors that may result in the solution remaining undetermined due to the model failing to converge [*Van den Meersche et al*., 2008].

### Covariance Terms

**Figure 6:**
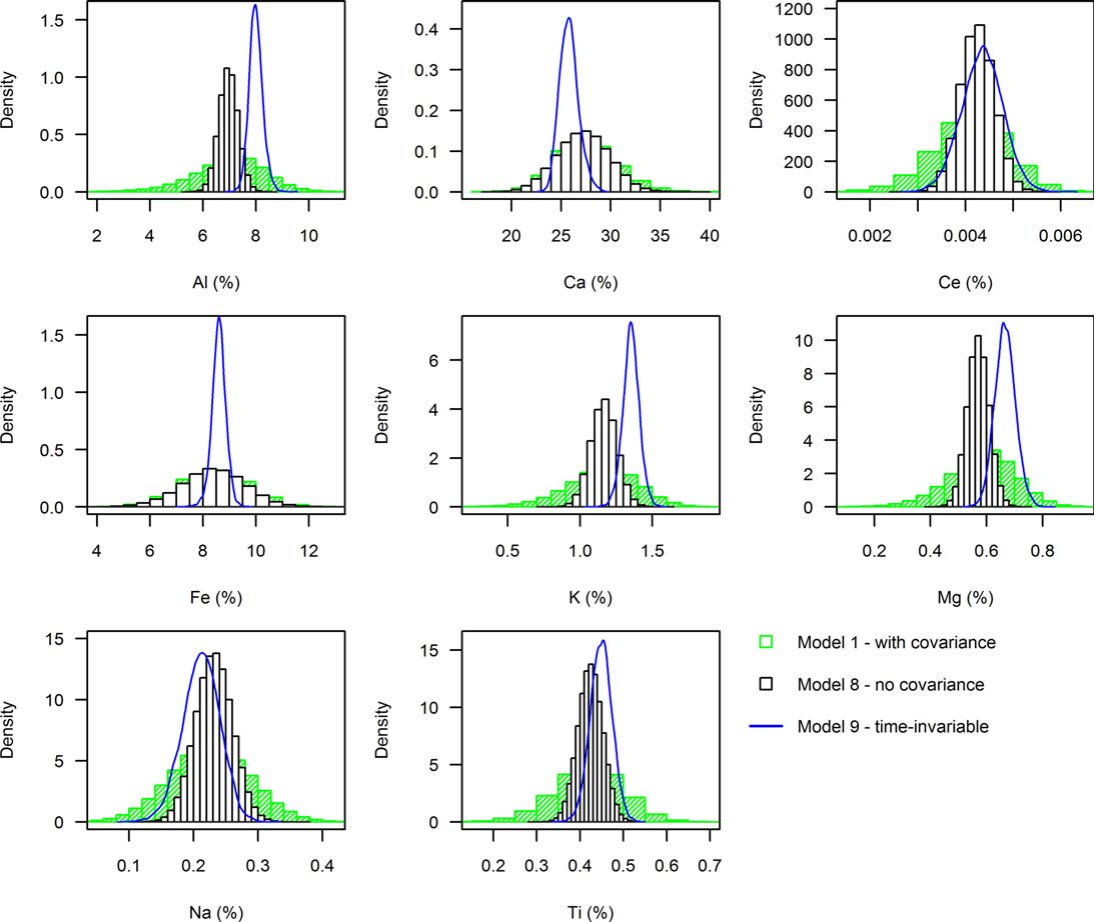
Histograms of the combined posterior subsurface source fingerprint concentrations for all 40 model time steps. Shown for models run with (M1) and without (M8) covariance terms, and with time-invariable source distributions (M9).

The considerable sensitivity demonstrated to the inclusion of covariance terms is consistent with previous studies [e.g., *Hopkins and Ferguson*, 2012; *Laceby and Olley*, 2014]. The narrowing of the uncertainty ranges around apportionment estimates is the result of the narrower source and combined residual-instrument error distributions that occur when covariance terms are omitted from the priors (Figure 6). This occurs most strongly for geochemical fingerprints that display a high degree of correlation, and thus covariation, with other elements (e.g., Pearson correlation between Al and Mg was 0.93 here). Thus, as the model degrees of freedom increase with the inclusion of covariance terms, the uncertainty around apportionment estimates also increase. Lower sensitivity is exhibited to the inclusion of residual-instrument error covariance because, as is often the case [*Phillips and Gregg*, 2001], instrument error is small in comparison with source area variability. However, by omitting covariance for either term, one is essentially stating that source fingerprint concentrations are linearly independent and that residual-instrument error is random and uncorrelated [*Christensen and Gunst*, 2004], conditions not satisfied by the data used here. This false assumption translates into unrealistically narrow uncertainty intervals around apportionment estimates. We can therefore state that whilst modeling of covariance can be difficult due to issues of dimensionality and positive-definiteness constraints [*Barnard et al*., 2000], its inclusion within mixing models is essential if posterior distributions are to accurately represent important codependencies between fingerprints [*Erhardt and Bedrick*, 2013]. Until now, such covariation has been largely ignored in sediment fingerprinting studies.

### Time Variability

The consequence of keeping source distributions fixed between time steps in M9 is that, over the 40 time steps, source area distributions were less variable than those observed in M1 (Figure 6). This also meant that there are fewer degrees of freedom in the model, thus limiting the choice of feasible values that could fit all time steps. Consequently, the location of proportion distributions was shifted and the uncertainty around apportionment estimates increased. It is also important to recognize that by omitting time-variable sources [e.g., *D'Haen et al*., 2012; *Erhardt and Bedrick*, 2013], mixing models are unable to account for important temporal factors, such as the transient delivery of sediment to the river channel, or the erodability and connectivity of different sediments at different times within each source area [*Fox and Papanicolaou et al*., 2008]. The approach taken in the benchmark model is therefore favored on mechanistic grounds as these influential variables are implicitly accounted for through more flexible posterior source distributions.

### Dirichlet Distribution and CLR Transformation

Out of all mixing model versions, sensitivity was lowest to the selection of either a Dirichlet distribution or a CLR-transformation to characterize the prior proportions. Whilst this implies any method is suitable, previous research has strongly indicated that geometric transformations are preferable [e.g., *Semmens et al*., 2009; *Parnell et al*., 2013]. This is because the compositional data of the proportions are in a closed form (i.e., must be positive and sum to unity) meaning that any increase in one source will inevitably result in a decrease in another source due to cross-dependencies, regardless of whether there is any mechanistic link between them [*Moore and Semmens*, 2008]. Under such circumstances, traditional statistical methods are not appropriate as they are designed to work in independent infinite space, not in situations where there are strong negative covariances between elements [*Pawlowsky-Glahn and Egozcue*, 2006]. Selection between the ILR and CLR transformations appears more subjective, although the ILR can be considered a more subcompositionally coherent transformation due to the orthogonal nature of the data [*Egozcue et al*., 2003].

### Full Bayes

The high sensitivity exhibited to the full Bayes approach arises because the fixed hyperparameters of M1 are replaced with more uninformative hyperparameters that are treated as random variables with prior distributions. This increases both the degrees of freedom and the variability of source distributions, thereby allowing a greater range of potential solutions. Whilst this sensitivity supports the results of *Ward et al*. [2010], who similarly found increased variability in source contributions when employing a full Bayesian approach, it is in contrast to *Parnell et al*. [2013] who found little difference between the two formulations. This seems to indicate that the implications of approximating full with empirical Bayesian methods are data specific and may vary between studies. The decision on which approach to adopt may come down to how much prior knowledge the modeler has on the system being studied, particularly in relation to how well catchment-wide variability in source area geochemistry is captured by the sediment samples obtained in the field. If source distributions are unrepresentative or mixing space geometry is poor, apportionment via the more flexible full Bayesian approach would likely prove more accurate [*Ward et al*., 2010]. However, such full methods increase model complexity and convergence times, and can lead to correlation between estimated proportions and estimated source means as the model updates prior distributions based on the information in the data [*Ward et al*., 2010]. Ultimately, the modeler will have to weigh up the trade-off between model accuracy and slow convergence (full Bayesian) and potential bias (empirical Bayes) [*Ward et al*., 2011].

### Frequentist Optimization

**Figure 7:**
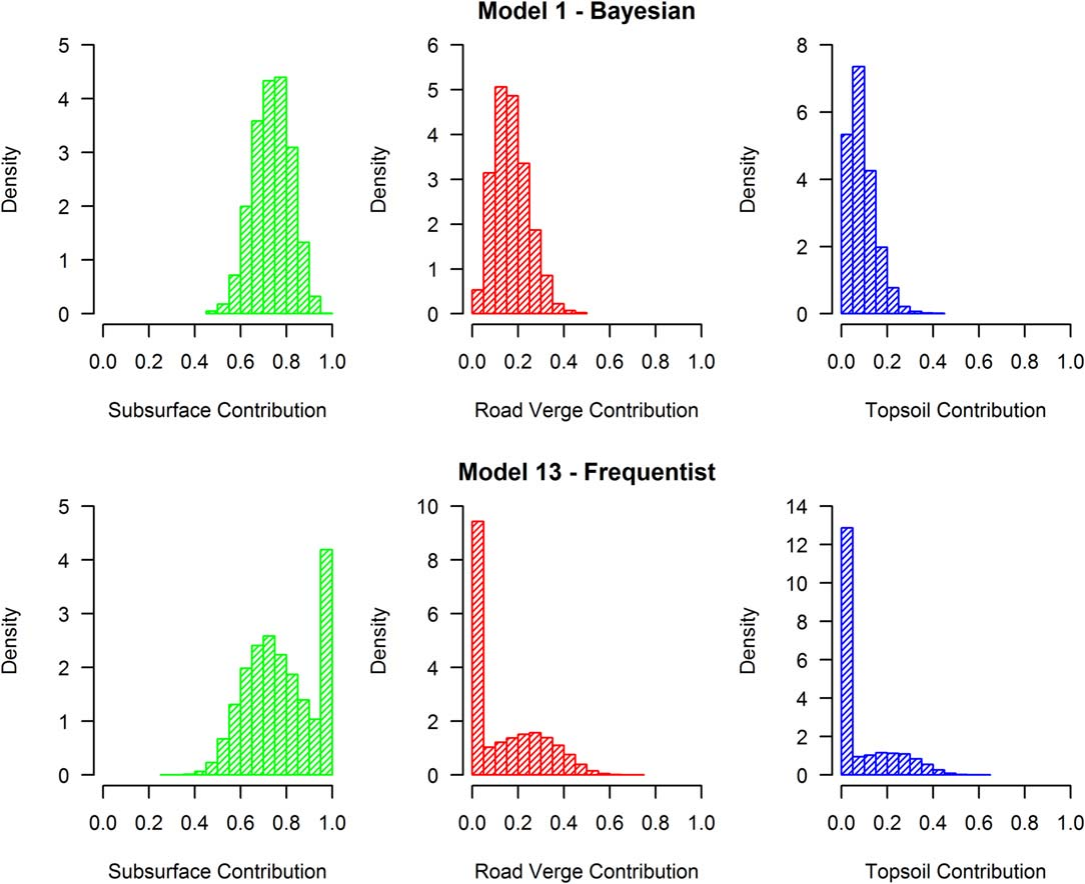
Comparison of Bayesian (M1) and frequentist (M13) source apportionment histograms for the first model time step, showing the heavily skewed distributions that result from the frequentist optimization within a Monte Carlo wrapper.

Large apportionment differences between frequentist and Bayesian approaches, which have similarly been recorded in other studies [e.g., *Nosrati et al*., 2014], can primarily be explained by the type of inference employed. The frequentist method employed here only carries out “point” optimization, whereby single random draws are made from the source and target distributions at each iteration of the Monte Carlo wrapper. It is therefore unable to yield full distributions for all of the underlying parameters. A direct consequence is that the optimization can produce heavily skewed proportion distributions whereby the best fit arises when one source supplies 100% of the sediment and the other sources supply 0% (Figure 7). Such skewed distributions are commonly seen in other fingerprinting studies that adopt similar pseudo uncertainty approaches to optimization [e.g., *Collins et al*., 2012] and it can lead to a high solution rejection rate by the GOF criterion, with as few as 10–20% of the solutions being accepted. This situation does not arise in Bayesian inference because the entire distributions of all parameters are fully explored together, resulting in more realistic posterior distributions [*Schmelter and Stevens*, 2013].

**Figure 8:**
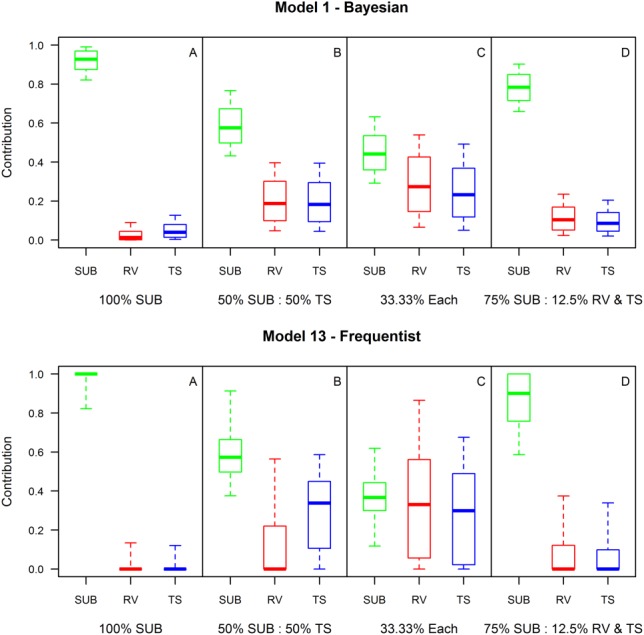
Comparing how Bayesian (M1) and frequentist (M13) mixing models perform in identifying sediment samples derived from (a) 100% subsurface sediment, (b) a 50:50 subsurface-topsoil mix, (c) a 33.33% mix of all three sources, and (d) a 75% subsurface, 12.5% road verge, 12.5% topsoil mix. The solid central line, boxes, and whiskers represent the median, 50%, and 95% credible intervals, respectively.

To further investigate the performance of the two approaches, M1 and M13 were re-run on four known laboratory mixtures (Figure 8). Namely, (Figure 8a) a pure subsurface sediment, (Figure b) a 50:50 subsurface-topsoil mix, (Figure 8c) a 33.3% mix of all three sources, and (Figure 8d) a 75% subsurface, 12.5% road verge, and 12.5% topsoil sediment mixture. The results show that whilst both models can unmistakably identify pure subsurface sediments (Figure 8a), the frequentist model yielded more accurate median contributions. This apparent unique solution might suggest superiority of the frequentist approach. However, as demonstrated in Figure 7, this is an artifact of point optimization within a Monte Carlo wrapper where 100% apportionment from one source is a common result. When the target samples fall between source regions within the mixing space, as occurs in reality and with the other mixtures shown here, the greater accuracy and precision of the Bayesian approach becomes clear once more, particularly with mixtures shown in Figures 8c and 8d.

### Implications and Recommendations

The results of this OFAT sensitivity analysis, which are supported by very similar findings from a four end-member mixing model for the neighboring minicatchment B (supporting information Figure S01), clearly demonstrate that differences in mixing model structure can impact significantly upon the resulting source apportionment estimates. Without exception, all model versions estimated subsurface sediment sources to be the major contributor of SPM to the River Blackwater under base flow conditions. However, there existed considerable variation in CI widths and estimated median proportions when one considers that all models used the same empirical data. Indeed, source apportionment results proved particularly sensitive to the selection of frequentist and full or empirical Bayesian approaches, especially with respect to the estimated uncertainty. Across the 13 model versions, median contributions (95% CI ranges) varied between 2.9 and 15.6% (14.5–36.6%) for topsoils, 1.8 and 22.9% (16.2–42.1%) for road verges, and 65.0 and 82.5% (13.8–48.2%) for subsurface sources, thus yielding a maximum median apportionment variation of 21.1% between models (Figure 5). To put this in context with other sources of fingerprinting uncertainty, *Smith and Blake* [2014] found that the commonly applied particle-size correction shifted mean source apportionment by 0–11% relative to the uncorrected model, whilst correcting for both organic matter content and particle size shifted mean apportionment by 0–45%. Similarly, *Laceby and Olley* [2014] found median source apportionment differences of 0–97% between models with or without tracer discriminatory and source variation weightings. It is therefore apparent that differences in mixing model structure play an equally important role in influencing source apportionment results as applying weighting factors or correcting for particle size and organic matter content. This has significant implications for the interpretation of results from other fluvial sediment fingerprinting investigations. If previous frequentist fingerprinting studies were to be repeated within a Bayesian framework, the results presented here indicate that apportionment results, and the conclusions based upon them, may be different. We therefore recommend that users of sediment fingerprinting mixing models carefully consider and justify their choice of model structure and error assumptions when conducting source apportionment studies. Specifically, we advocate a Bayesian approach to mixing model formulation as it provides a robust and flexible framework in which all known and residual uncertainties associated with the mixing model and the data set can be fully and coherently translated into parameter probability distributions. Furthermore, we recommend the inclusion of covariance terms to ensure that models accurately represent codependencies between selected fingerprints and thereby minimize the risk of obtaining unrealistic uncertainty intervals around proportion estimates. We also advise that source distributions are time variable to enable models to account for differences in source area erodability, connectivity, and transient sediment storage. Mechanistic extensions of the mixing model to account for the same effects should be an area of further research, but would require more data for parameterization. Finally, we recommend that, where possible, uninformative hyperparameters should be used within a full Bayesian framework to stay true to the Bayesian paradigm and minimize the risk of unrepresentative data-biasing results, particularly when fingerprints are poorly defined. However, we acknowledge that convergence issues can arise when model priors are set too vague, and therefore empirical Bayes methods can provide a pragmatic approximation.

## Conclusions

The sensitivity of fluvial sediment source apportionment estimates to changing mixing model structure has been assessed via a one-factor-at-a-time (OFAT) sensitivity analysis. Thirteen model versions were developed, each with slightly different structures and error assumptions. All 13 models were then applied to SPM geochemistry data from the River Blackwater, Norfolk, UK, to apportion sediment contributions from three sources (arable topsoils, road verges, and subsurface sediments) under base flow conditions for the period August 2012 to August 2013. Whilst all models estimated subsurface sediments to be the largest contributor of SPM (median ∼76%), comparison of apportionment estimates across model versions reveals varying degrees of sensitivity to changing priors, inclusion of covariance terms, incorporation of time-variant distributions, and methods of proportion characterization. In particular, we have demonstrated substantial differences in apportionment results between full and empirical Bayesian approaches, and between Bayesian and frequentist frameworks, with median apportionment varying by up to 21% between model versions. Mixing model structure thus impacts heavily upon the resulting source apportionment estimates. This has notable implications for the interpretation of results from other sediment fingerprinting investigations which, due to the lack of a coherent modeling framework, employ a wide variety of modeling approaches that often incorporate source and target uncertainty ad hoc. We therefore conclude that users of sediment mixing models should fully consider what impact their choices of model structure and error assumptions have on the resulting source apportionment estimates prior to conducting sediment fingerprinting investigations.

## Supporting Information

Readme

Figure S01

Benchmark model
