# Supplementary material for: Sensitivity of fluvial sediment source apportionment to mixing model assumptions: A Bayesian model comparison
Source: Water Resour Res. 2014 Nov 21;50(11):9031–47. doi: 10.1002/2014WR016194 (PMC4650832; doi:10.1002/2014WR016194)
Supplement: Supplementary file 1 — Readme [file wrcr0050-9031-sd1.docx]

Auxiliary Material for:

**Sensitivity of Fluvial Sediment Source Apportionment to Mixing Model Assumptions: A Bayesian Model Comparison**

Richard J. Cooper^1^, Tobias Krueger^2^, Kevin M. Hiscock^1^, and Barry G. Rawlins^3^

^1^School of Environmental Sciences, University of East Anglia, Norwich Research Park, Norwich, NR4 7TJ, UK

^2^ IRI THESys, Humboldt University, 10099 Berlin, Germany

^3^ British Geological Survey, Keyworth, Nottingham, NG12 5GG, UK

Water Resources Research, 2014

**Introduction**

This supplementary material contains the code “Benchmark_Model”, in .txt format, required to run the benchmark Bayesian mixing model (M1) through JAGS in the R environment. This code represents a modified version of that developed by Parnell *et al*. [2013]. Also included is figure “fs01.tiff” which presents OFAT sensitivity analysis results for a four end-member mixing model from the neighboring mini-catchment B in support of Figure 5.

**Figure Captions**

**Figure S01**: Comparison of SPM source apportionment for mini-catchment B as estimated by 13 four end-member mixing model versions. Results for each version are displayed as the temporal apportionment average across 10 baseflow SPM samples spanning May-September 2012. Points represent median contributions with associated 95% credible intervals, whilst dashed lines represent the median contribution estimated by M1. Note how the inter-model variability in median source apportionment and 95% credible interval width closely matches the trends observed with data from mini-catchment A (Figure 5).
