# Supplementary figures and images for: Sensitivity of fluvial sediment source apportionment to mixing model assumptions: A Bayesian model comparison
Source: Water Resour Res. 2014 Nov 21;50(11):9031–47. doi: 10.1002/2014WR016194 (PMC4650832; doi:10.1002/2014WR016194)

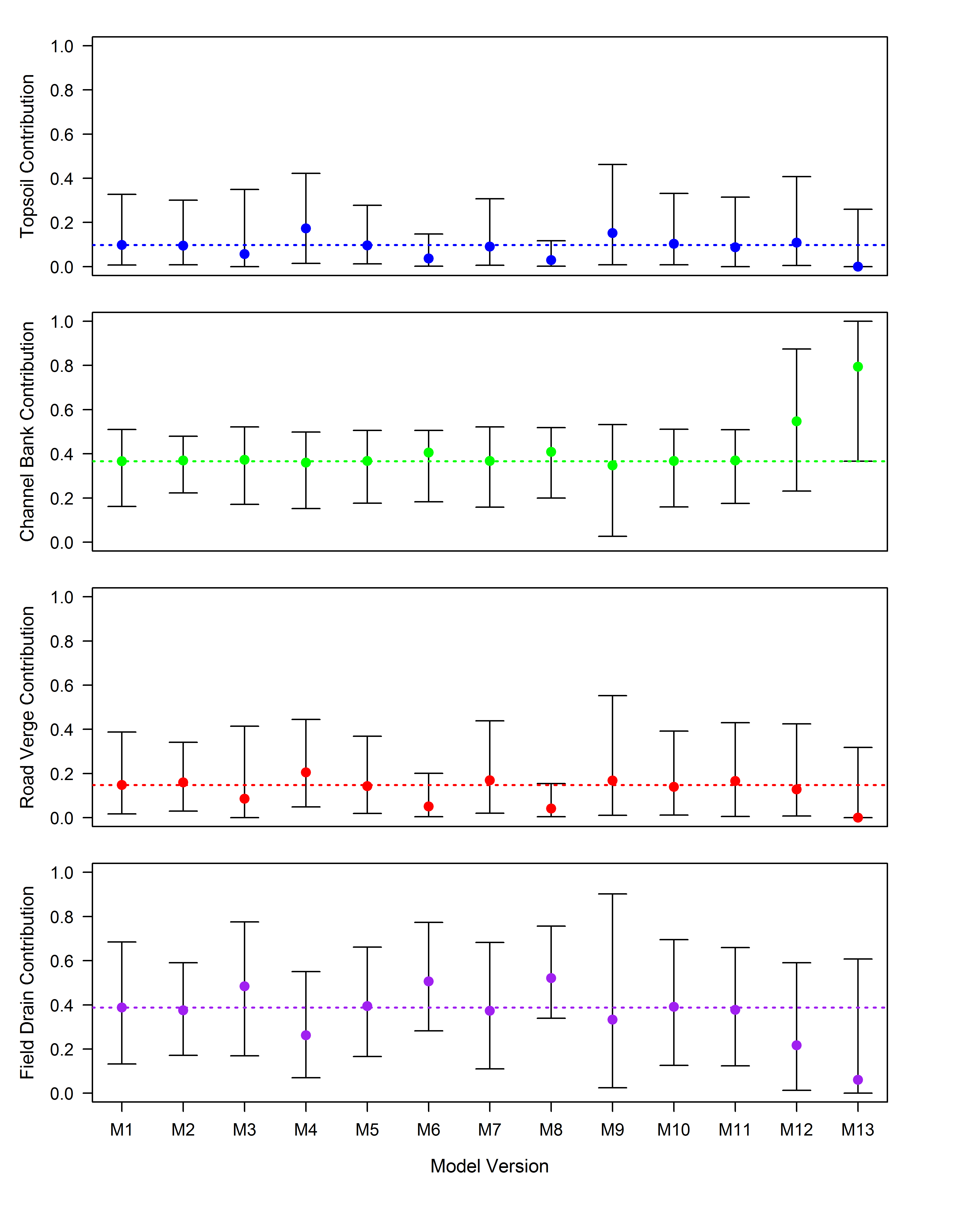

Supplement: Supplementary file 2 — Figure S01 [file wrcr0050-9031-sd2.tiff]
